# Pre-clinical efficacy evaluation of human umbilical cord mesenchymal stem cells for ischemic stroke

**DOI:** 10.3389/fimmu.2022.1095469

**Published:** 2023-01-13

**Authors:** Danpeng Shen, Hongwei Wang, Hongyan Zhu, Cuibao Jiang, Fan Xie, Hongpeng Zhang, Qian Lv, Qi Liu, Zhiqiang Wang, Nianmin Qi, Hao Wang

**Affiliations:** Research and Experimental Development Department, Asia Stem Cell Regenerative Pharmaceutical Co. Ltd, Shanghai, China

**Keywords:** hUCMSCs, MCAO, Ischemic stroke, Time windows, Dose-effect relationship

## Abstract

**Objective:**

This study explored the underlying therapeutic mechanism of human umbilical cord mesenchymal stem cells (hUCMSCs) for ischemic stroke (IS), and determined the optimal administration time windows and dose-effect relationship.

**Methods:**

The levels of SDF-1α, IL-10, IL-6, TNF-α, BDNF, IL-1β, and VEGF-A factors in serum and brain tissue lysate were measured by ELISA. The pathological status of brain tissues was evaluated by Hematoxylin-Eosin (HE) staining, and apoptosis of nerve cells was detected by tunel. The protein expression of CXCR-4, NeuN, and Nestin in the brain tissues was assessed through immunofluorescence. The balance beam, forelimb muscle strength, and limb placement were tested on MCAO rats at different time points and doses. The infarct area of the rat brain tissues was measured at the end of the experiment.

**Results:**

The hUCMSC treatment during the acute phase of MCAO significantly reduced the secretion of IL-6, TNF-α, IL-1β but increased IL-10 in serum, and the levels of SDF-α and BDNF in serum and brain tissues lysate were also increased. The pathological results showed that there were more neurons in the treatment group compared to the model group. Immunofluorescence assays showed that the expression of CXCR4、Nestin、NeuN was relatively higher than that in the model group. The d4 and d7 treatment significantly improves the motor function, promotes the recovery of forelimb muscle strength, increases the forelimb placement rate and reduces the scope of cerebral infarction, but the d14 treatment group has less therapeutic effect compared to the d4 and d7 treatment. The 2×10^7^/kg treatment showed the best therapeutic effect, followed by the 1×10^7^/kg treatment, and the worst is 0.5×10^7^/kg treatment from the test of balance beam, forelimb muscle strength, limb placement and the infarct area of the rat brain tissues.

**Conclusion:**

The hUCMSCs can inhibit the infiltration of inflammatory cells in the brain tissue, and promote the repair of brain tissue structure and function. Early intervention by injecting high-dose of hUCMSCs can significantly improve the recovery of neurological/motor function and reduce the size of cerebral infarction in rats.

## 1 Introduction

Stroke is a destructive cerebrovascular event caused by the interruption of cerebral blood flow due to a blockage or burst/hemorrhage of cerebral vessels (hemorrhagic stroke; HS), which led to physical disability and multiple secondary impairments of physical function (1). The Guidelines for the Prevention and Treatment of Stroke in China (2021 Edition) defines the onset of stroke within 2 weeks as the acute phase, between 2 weeks and 6 months as the recovery phase, after 6 months as the sequelae phase, and after 2 weeks also regarded as the sub-acute phase (2, 3). Furthermore, the latest Global Burden of Disease Study (GBD) shows that the overall lifetime risk of stroke in China is 39.9%, ranking the first in the world, which means that about two out of every five chinese will suffer from stroke in older adults over 75 (4). In addition, stroke is also the top cause of death among all diseases in China (5, 6).

The middle cerebral artery (MCA) is a vulnerable site for stroke, and the middle cerebral artery occlusion (MCAO) model is generally accepted as the standard animal model for focal cerebral ischemia (7, 8). The basic principle involves the occlusion of the middle cerebral artery origin with a wire plug, causing the middle cerebral artery feeding area to be ischemic for a certain time, which in turn leads to the rats developing focal cerebral ischemia (9). This method is characterized by good stability, reproducibility, small injury, exact infarct location, and high success rate (8). Therefore, this model was adopted in this study to simulate human stroke and conduct drug treatment studies (10).

Umbilical cord mesenchymal stem cells (UC-MSCs) are a type of fibroblast-like cells present in the fahrenheit gum and are mesoderm-derived multipotent stem cells with high self-renewal capacity and multilineage differentiation potential (11). hUCMSCs can be induced to differentiate into neural stem cells *in vitro*, and they still exist and express neuron-specific markers three weeks after direct brain transplantation (12), and hUCMSCs can cooperate with some other drugs such as neural stem cells or corresponding culture medium to produce a better therapeutic effect (13). hUCMSCs have an important application value in improving the clinical symptoms of IS patients, and studies have shown that UC-MSCs inhibit lesion symptoms in the acute phase and promote cerebral ischemic site repair in the chronic phase through different mechanisms (14). During IS treatment, hUCMSCs migrate to the location of the hypoxia-induced brain injury *via* the homing effect mediated by stromal cell-derived factors and chemokine receptors and exert their functions through immunomodulation, anti-inflammation, inhibition of apoptosis, vascular regeneration, neural repair, and remodeling (3, 15–18). Although numerous pre-clinical and clinical studies on the use of stem cells in stroke have reported their safety and effectiveness, problems regarding the pre-clinical treatment time windows, dosage, and mechanism of preparation of hUCMSCs in a mature system still need to be clarified (19–22). In this study, a pharmacodynamic evaluation of hUCMSCs for the treatment of IS was performed to determine its therapeutic mechanism, different time windows, and the dose-effect relationship.

## 2 Methods

### 2.1 Preparation of the hUCMSCs

The hUCMSCs were obtained from Shanghai Quansheng Biotechnology Co., Ltd., which were extracted and cultured from the umbilical cords of newborns. The umbilical cords were obtained from Dongguan Chang’an Hospital and approved the inspection of the ethics committee of the hospital. hUCMSCs were routinely tested for surface markers, three-line differentiation function, immunomodulatory function, fungi, bacteria, and other indicators before use. hUCMSCs were approved for use after meeting the certificate of analysis (COA) release criteria.

### 2.2 Model establishment

The use of experimental animals was approved by the Laboratory Animal Ethics Committee of Youji (Tianjin) Pharmaceutical Technology Co., Ltd. In this experiment, the rats were anesthetized with isoflurane and fixed in the supine position. The skin was incised along the median line of the neck to expose the right common carotid artery. The nerves and fascia around the blood vessels from the bifurcation of the common carotid artery to the skull base were carefully removed. A nylon suture (Diameter 0.28 mm) was introduced into the internal carotid artery from the distal end of the external carotid artery and inserted into the circle of Willis at the middle cerebral artery to effectively blocks the middle cerebral artery. The length of the inserted suture was 18 to 20 mm from the bifurcation of the common carotid artery. The free end of the external carotid artery was then ligated with an intraluminal suture to prevent bleeding. The subcutaneous fascia and skin were sutured layer by layer, and penicillin was dripped locally to prevent infection. In the control group, only the internal carotid artery was separated. Two hours after the start of MCAO, the thread in the lumen of the internal carotid artery was carefully extracted to reperfuse the artery, followed by feeding the animals.

The Neurological impairment degree score (NDS) was assessed after 2 hours of ischemia and reperfusion (23). A point above 8 meant a successful model (24). Samples with 10-12 points were selected for downstream experiments. The detailed scoring criteria are indicated in **Supplementary Table 1**.

### 2.3 Experimental design

**Figure 1:**
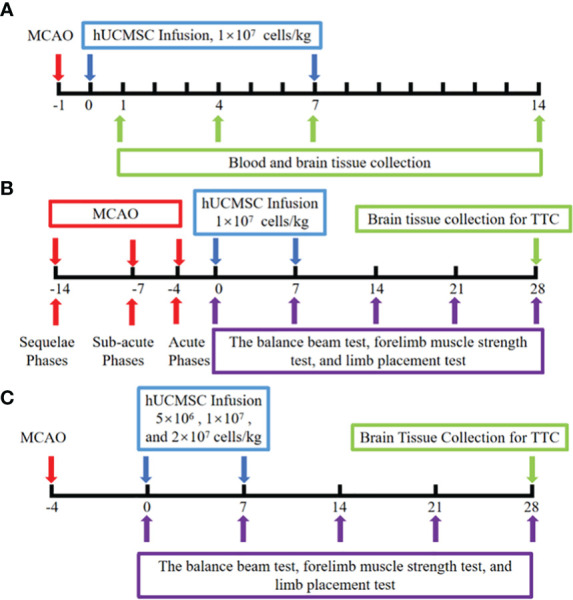
Schematic illustration of hUCMSCs treatment for ischemic stroke experiment. **(A)** The mechanism experiment; **(B)** The time windows experiment; **(C)** The dose-effect relationship experiment; The red arrows indicate the model establishment time points; The blue arrow indicates the time point of injection of hUCMSCs or PBS; The purple arrows indicate the time points of motor/neural function testing; The green arrow indicates the time point of sample collection and detection.

In the mechanism experiment, hUCMSCs were injected into the rat tail vein at a dose of 1 mL/kg based on the standard of 1×10^7^ cells/ml on d1 after modeling. After 7 days, the second administration was carried out. The model group were given the same dose of PBS. Each group consisted of 12 rats, and the serum and brain tissues of MCAO rats on the d1, d4, d7, and d14 were collected after hUCMSCs or PBS infusion. Normal rat blood and brain tissue lysates were used as initial control data before the experiment was carried out. The protein levels of SDF-1α, IL-10, IL-6, TNF-α, BDNF, IL-1β, and VEGF-A in serum and brain tissue lysates were measured using Luminex multi-factor kits (R&D). Meanwhile, the protein levels of CXCR-4, NeuN, and Nestin in the brain tissues of d14 rats were determined by immunofluorescence. The pathological structures in the brain tissues were examined by HE staining (**Figure 1A**).

In the time window experiments, the hUCMSCs were injected into the rat tail vein on d4, d7, and d14 after MCAO, corresponding to the acute, sub-acute, and sequelae phases of IS, respectively. The dose of hUCMSCs was 1×10^7^cells/kg, and the second treatment was performed 7 days after the first administration. Each time point was divided into control group, model group and treatment group with 10 rats in each subgroup. The clinical observation period for the rats lasted until 28 days after the first administration. The balance beam test, forelimb muscle strength test, and limb placement test were all performed on the rats before administration and on the 7d, 14d, 21d, and 28d after administration. The infarct area was detected by triphenyl tetrazolium chloride (TTC, Phygene) staining at the end of the experiment (**Figure 1B**).

Based on the results of the time window experiments, the optimum time point for the injection of hUCMSCs was determined, and the dose-effect relationship experiment was carried out. The low-dose, medium-dose, and high-dose hUCMSCs treatment groups were set up, with doses of 5×10^6^ cells/kg, 1×10^7^ cells/kg, and 2×10^7^ cells/kg, respectively, with 12 rats in each group. The balance beam test, forelimb muscle strength test, and limb placement test were performed on 7d, 14d, 21d, and 28d after the first administration, and the cerebral infarction area was detected by TTC staining at the end of the experiment (**Figure 1C**).

### 2.4 Motor function scoring

The balance beam, forelimb placement, and forelimb muscle strength of MCAO rats were tested before the first administration and on 7d, 14d, 21d, and 28d after administration to evaluate the recovery of neurological/motor function of MCAO rats (25).

### 2.5 Determination of the cerebral infarction area

Following the motor function experiments, the rats were sacrificed by cervical vertebra detachment after isoflurane gas anesthesia and bleeding. The brain tissues were harvested, stored in a -80°C freezer, and then sectioned from front to back with a thickness of 2 mm. Brain tissue sections were placed in 2% red tetrazolium solution and incubated at 37 °C for 5 minutes. Infarct tissues were white and non-infarct tissues were red. The cerebral infarction area (%) was measured using Image J software. Bias due to brain edema or brain atrophy was excluded using a modified formula as follows:

Cerebral infarction area (%) = (area of the hemisphere on the non-infarct side – area of normal brain tissue on infarct side)/(area of the hemisphere on non-infarct side × 2)× 100% (26, 27).

### 2.6 Data analysis

The SPSS 22 software was used for statistical analysis. One-way analysis of variance (ANOVA) was used for multi-group comparison. Meanwhile, the independent data of the two groups were analyzed using a t-test. A P<0.05 was considered statistically significant. The graphical representations of the results were constructed using the Graphpad Prism 8 software.

## 3 Results

### 3.1 Phenotype and multilineage differentiation ability validation

**Figure 2:**
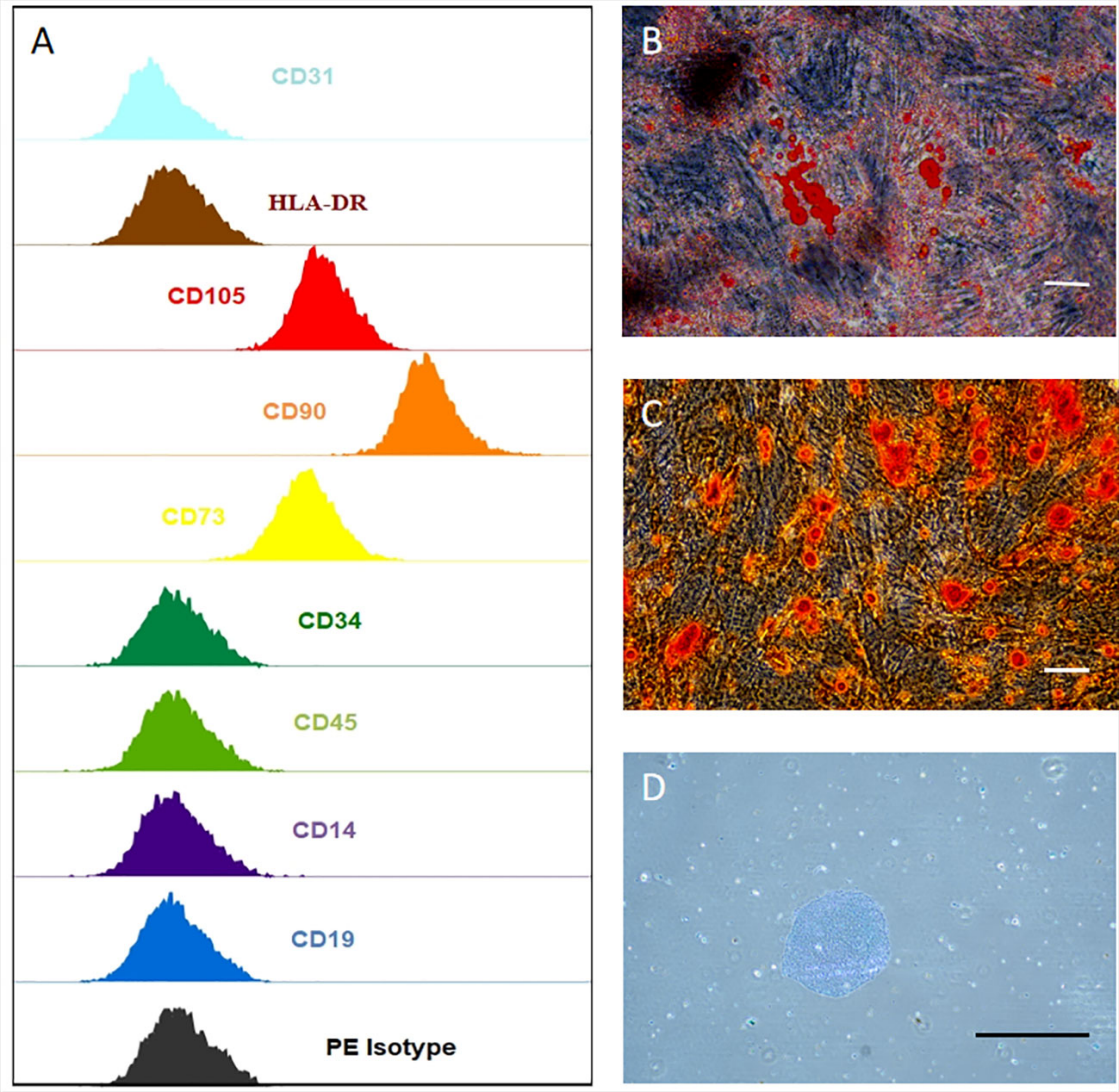
hUCMSCs surface markers detected by flow assay and three-lineage (adipogenic, osteogenic, chondrogenic) differentiation ability detected by immunocytochemistry. **(A)** the negative expression of surface markers CD14, CD19, CD31, CD34, CD45, HLA-DR of hUCMSCs; and the positive expression of surface markers CD73, CD90, CD105 of hUCMSCs, antibody binding dye is PE; **(B)** Oil red O staining to analyze adipogenic differentiation of hUCMSCs; **(C)** Alcian blue staining to explore chondrogenic differentiation of hUCMSCs; D: Alizarin red staining to analyze osteogenic differentiation of hUCMSCs.The scale bar = 50 μm in **(B)** and 200 μm in **(C, D)**.

Cell surface markers of hUCMSCs were detected by flow cytometry assay, and the result indicates that they are CD14, CD19, CD31, CD34, CD45, HLA-DR negative and CD73, CD90, CD105 positive (**Figure 2A**). hUCMSCs were detected by immunocytochemistry for their lipogenic, osteogenic, and chondrogenic differentiation potential in the different induction differentiation media. The results showed that hUCMSCs could also successfully transdifferentiate into adipocytes (analyzed by Oil Red O staining), osteoblasts (analyzed by Alizarin Red staining), and chondrocytes (analyzed by Arsenic Blue staining) (**Figures 2B–D**). Together, these results indicate that hUCMSCs are characterized by high purity and with multidirectional differentiation capabilities.

### 3.2 Administration of hUCMSCs affected the levels of cytokines in the blood and brain tissue lysates in MCAO rats

**Figure 3:**
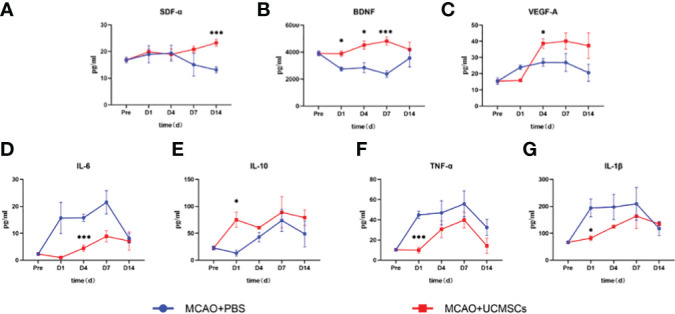
Cytokine levels in serum samples of MCAO rats after administration of hUCMSCs. **(A–G)** Expression levels of SDF-α, BDNF, VEGF-A, IL-6, IL-10, TNF-α, and IL-1β in serum, respectively. Data are presented as mean ± SD. Statistically significant values between the treatment and the model groups are denoted as *P < 0.05, and ***P < 0.001.

**Figure 4:**
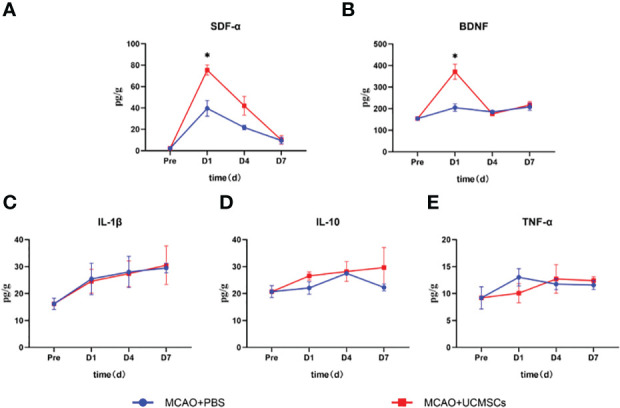
Cytokine levels in the brain tissue lysates of MCAO rats after administration of hUCMSCs. **(A–E)** Expression levels of SDF-α, BDNF, IL-1β, IL-10, and TNF-α in brain tissue lysates, respectively. Data are presented as mean ± SD. Statistically significant values between the treatment and the model groups are denoted as *P < 0.05.

The administration of hUCMSCs led to a significant reduction in the levels of the proinflammatory factors IL-1β, IL-6, and TNF-α and an increase in the levels of the anti-inflammatory factor IL-10 in serum (**Figures 3D–G**), but no significant difference in the levels of these factors has been observed in the brain tissue samples (**Figures 4C–E**). Furthermore, the hUCMSCs effectively promoted the secretion of the SDF-α, BDNF, and VEGF in the serum (**Figures 3A–C**). In addition, the levels of SDF-α and BDNF in the brain tissues also showed an increasing trend (**Figures 4A, B**); particularly, these factors were relatively high in the sub-acute phase of MCAO. Unfortunately, the levels of IL-6 and VEGF-A were lower than the sensitivity limit of the Luminex multi-factor kits. Based on these results, the hUCMSCs administration showed a good immunomodulatory function in the treatment of IS, improved the secretion of chemokines and neurotrophic factors in the sub-acute phase.

### 3.3 hUCMSCs administration promoted the recovery of the pathological structure of brain tissue and reduced apoptosis in MCAO rats

**Figure 5:**
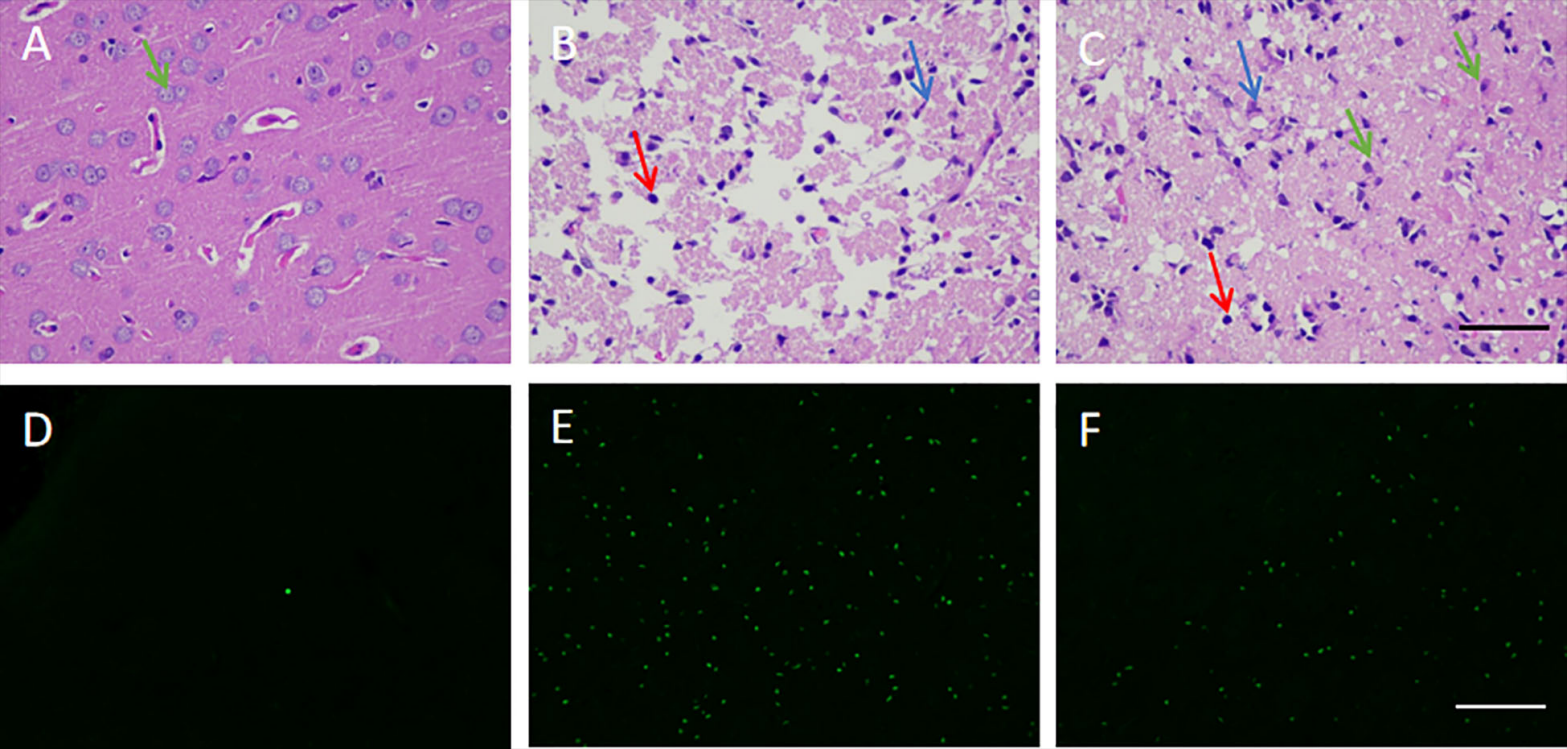
Effect of hUCMSCs on the pathological structure and apoptosis of brain tissues of MCAO rats. **(A–C)** HE staining results of the normal, model, and treatment groups on d14 after administration, respectively. The staining magnification is 400 times and the scale is set at 2.5 µm (green arrow: neuronal cells; red arrow: microglial cells; blue arrow: apoptotic neuronal cells); **(D–F)** tunel staining results of the normal, model, and treatment groups on d14 after administration, respectively. The staining magnification is 400 times and the scale is set at 2.5 µm.

The pathological and structural analysis of rat brain tissues in each group by HE showed that the normal control group had an intact brain structure with clear borders and abundant cell numbers (**Figure 5A**), while the model group had increased neuronal death and inflammatory cells, as well as vacuolar degeneration and edema formation (**Figure 5B**). Meanwhile, the treatment group was characterized by relatively more neuronal cells, regular brain tissue and cell arrangement, and a more complete structure (**Figure 5C**). Compared with the model group, the striatum of the treatment group showed a smaller number of apoptotic cells (**Figures 5D–F**). These results indicate that hUCMSCs administration *via* the tail vein could effectively inhibit pathological damage, reduce inflammatory infiltration, and promote tissue repair to some extent in the brain tissues of MCAO rats.

### 3.4 hUCMSCs administration increased the levels of chemokines and neuronal repair in the brain of the MCAO rats

**Figure 6:**
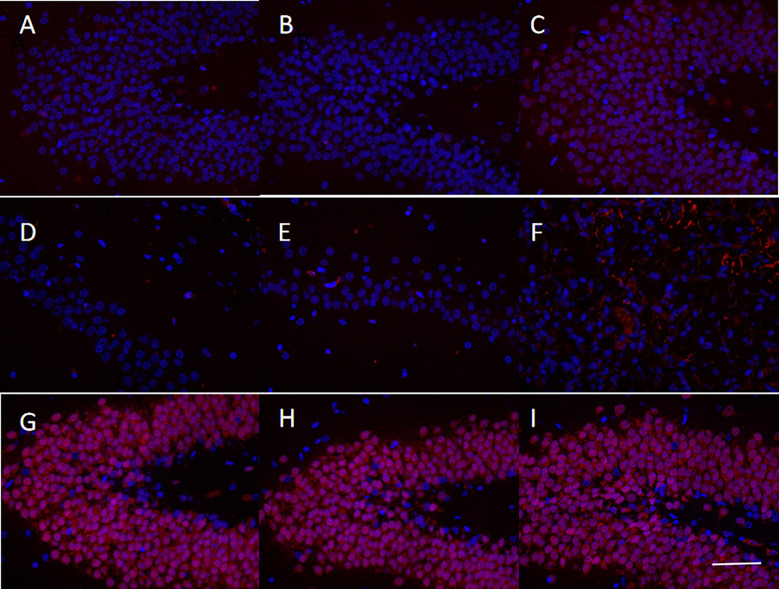
Levels of chemokines and neuronal markers in the brain tissues of MCAO rats after administration of hUCMSCs. The figures show the immunofluorescence staining results of CXCR4 **(A–C)**, Nestin **(D–F)**, and NeuN **(G-I)** in the normal group, model group, and treatment group, respectively. The staining magnification is 400 times and the scale is set at 2.5 µm.

Immunofluorescence was used to evaluate the protein expression of CXCR4, Nestin, and NeuN in the brain tissue of MCAO rats in the sequelae phase (**Figure 6**). Upon hUCMSCs administration in sequelae phase MCAO rats, an apparent upregulation of CXCR4 was observed in the treatment group compared with the model group (**Figures 6A–C**). The expression of Nestin, a neural stem cell marker (28), was significantly increased after the injection of hUCMSCs (**Figures 6D–F**). Consistently, the expression of NeuN, a neuronal marker (29), also showed an upward trend (**Figures 6G–I**). These findings suggest that the hUCMSCs administration could promote the expression of chemokines in the brain tissue of MCAO rats at the sequelae phase and promote the repair, proliferation, and maturation of neurons.

### 3.5 hUCMSCs administration improved motor function recovery and reduced cerebral infarction area in MCAO rats at different time windows

**Figure 7:**
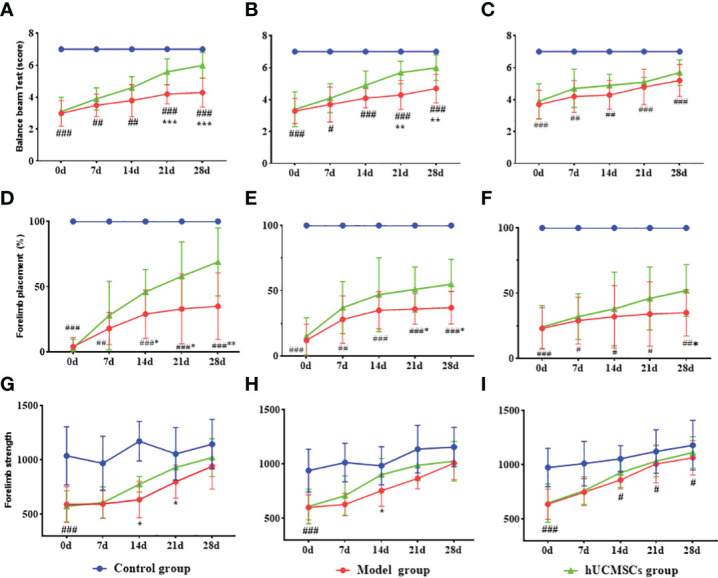
Effect of hUCMSCs administration on the neurologic/motor recovery of MCAO rats at different administration time points. **(A–C)** Balance beam test scores after administration on d4, d7, and d14 post-modeling. **(D–F)** Forelimb placement scores after administration on d4, d7, and d14 post-modeling. **(G–I)** Forelimb muscle strength scores after administration on d4, d7, and d14 post-modeling. Data are presented as mean ± SD. ^#^P < 0.05, ^##^P < 0.01, and ^###^P < 0.001 vs control group; ^**^P < 0.01, ^***^P < 0.001 vs model group.

The MCAO rat models were monitored for totally 28 days from the first injection of the hUCMSCs. Each experimental group showed motor function, balance beam crawling disorder, gait side-slip, forelimb placement rate, and forelimb muscle strength reduction (**Figures 7**). The d4 and d7 hUCMSCs treatment could significantly improve the motor function and forelimb placement rate from d21 to d28 after administration, and the gait side-slip was significantly reduced (**Figures 7A, B, D, E**). The d14 hUCMSCs treatment had a relatively weak lower trend in improving the balance beam test scores and forelimb placement rate (**Figures 7C, F**). The d4, d7, and d14 hUCMSCs treatment had significantly improved forelimb muscle strength (**Figures 7G–I**). Based on the results of the balance beam test, forelimb placement test, and forelimb muscle strength test, the d4 hUCMSCs treatment group showed a greater trend of score improvement in each group. Meanwhile, the d7 hUCMSCs treatment group had a slight score improvement, but the difference was relatively lower than that of the d4 hUCMSCs treatment group. In contrast, there was only a statistically significant difference in the forelimb muscle strength scores of the d14 hUCMSCs treatment group. These findings suggest that the hUCMSCs administration can promote the recovery of motor function in the MCAO model, with earlier intervention treatment time leading to a better therapeutic effect.

**Figure 8:**
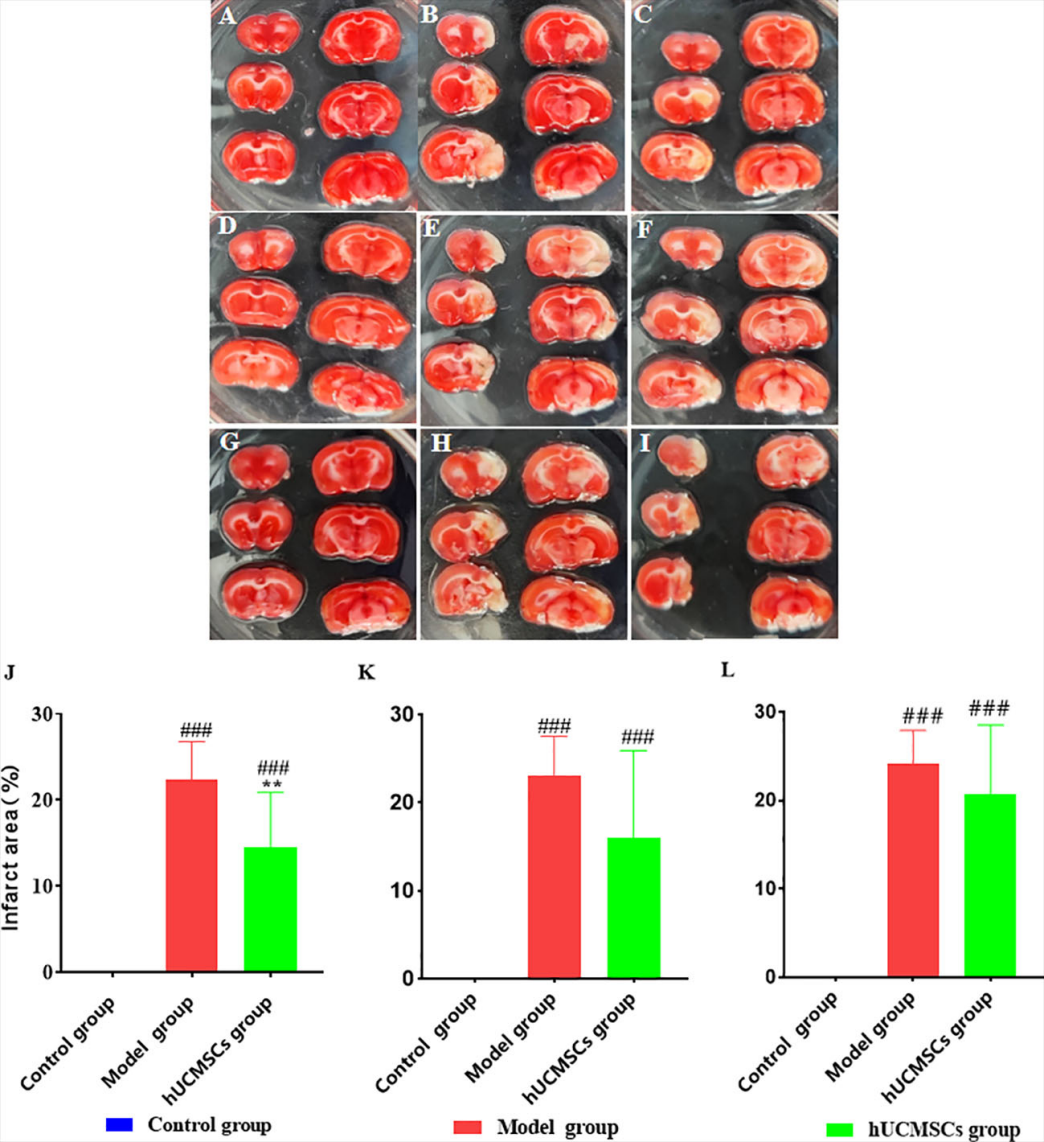
Effect of hUCMSCs administration on the size of the cerebral infarction area of MCAO rats at different administration time points. The figures show the results of the TTC staining of the MCAO rats’ brain tissue 28 days after administration. The white area means necrotic area, **(A–C)** The TTC staining of the control group, model group, and hUCMSCs treatment group after administration on d4; **(D–F)** The TTC staining of the control group, model group, and hUCMSCs treatment group after administration on d7; and **(G-I)** The TTC staining of the control group, model group, and hUCMSCs treatment group after administration on d14; **(J–L)** The proportions of cerebral infarction area as evaluated by TTC after administration on d4, d7, and d14 post-modeling. Data are presented as mean ± SD. ^###^P<0.001 vs control group; ^**^P<0.01 vs model group.

The brain tissues of rats in each group were stained with TTC 28 days after hUCMSCs administration (**Figures 8**). In the model establishment, the right cerebral infarction area of the MCAO rats was larger by inserting the suture from the right common carotid artery. The brain tissues in the model group and the treatment group showed a white cerebral infarction area, which was analyzed by the Image J software (**Figures 8A–I**). Compared with the model group, The d4 (**Figures 8B, C**), d7 (**Figures 8E, F**), and d14 (**Figures 6H, I**) hUCMSCs treatments showed less white areas after modeling. No white necrosis area in the control group at each time point has been observed (**Figures 8A, D, G**). In contrast with the model control group, the cerebral infarction area was reduced in the hUCMSCs treatment group on d4, d7, and d14 post-modeling, and the improvement rates were 35%, 30.4%, and 14.1%, respectively (**Figures 8J–L**). These findings indicate that within a certain time limit, early hUCMSCs treatment could reduce the cerebral infarction area and improve the therapeutic effect.

### 3.6 Different doses of hUCMSCs improved motor function recovery and reduced cerebral infarction area in MCAO rats

**Figure 9:**
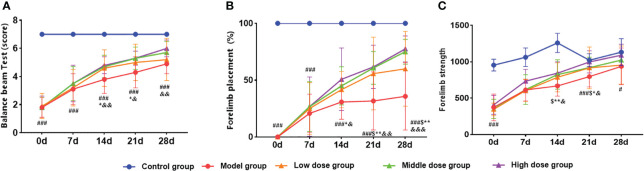
Effect of different doses of hUCMSCs on the recovery of neurological/motor function in MCAO rats. **(A–C)** Balance beam test, forelimb placement test, and forelimb muscle strength test results. Data are presented as mean ± SD. ^#^P<0.05, ^###^P<0.001 vs control group; ^$^P<0.05 low dose group vs model group; *P < 0.05, **P < 0.01 middle dose group vs model group; ^&^P < 0.05 high dose group vs. model group.

The time windows experiment showed that the hUCMSCs had a better therapeutic effect in acute IS. Based on this observation, the dose effect of the hUCMSCs was performed 4 days after modeling. In the balance beam test, the low-dose hUCMSCs treatment had no significant improvement effect (**Figure 9A**). Furthermore, the low-dose, medium-dose, and high-dose stem cell treatment could significantly improve the motor function of rat models on d14 to d21 after administration, while the gait side-slip was significantly reduced (**Figure 9A**). Additionally, the frequency of forelimb placement and forelimb muscle strength was significantly increased in all treatment groups (**Figures 9B, C**). There was a significant dose-dependent improvement in the comprehensive scores of neurological/motor functions in the high-dose treatment group compared with the medium-dose and low-dose treatment groups.

**Figure 10:**
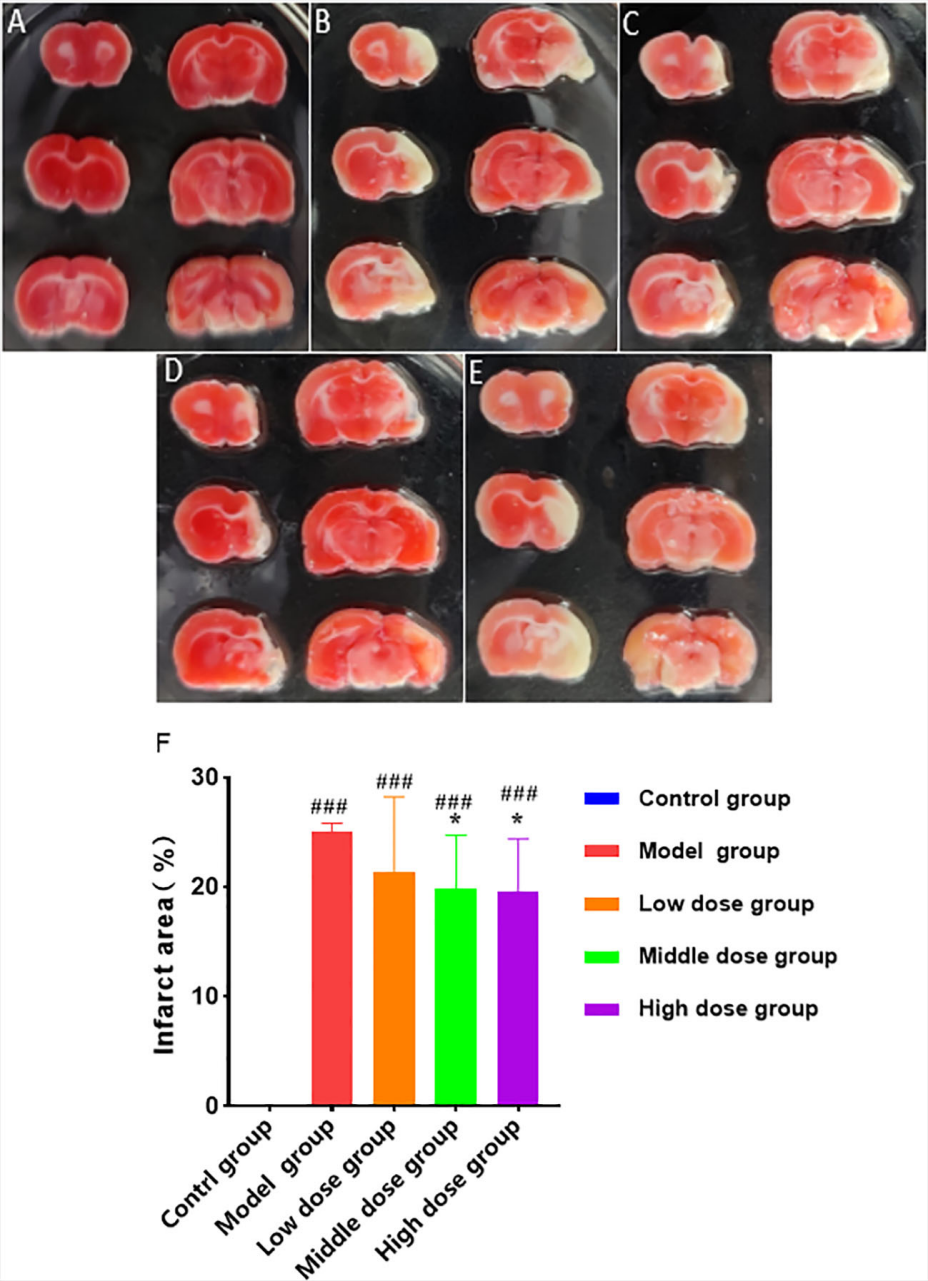
Effect of different doses of hUCMSCs on cerebral infarction area of MCAO rats. **(A–E)** Results are shown for the control group, model control group, low-dose hUCMSCs treatment group, medium-dose hUCMSCs treatment group, and high-dose hUCMSCs treatment group, respectively. **(F)** The proportion of cerebral infarction area in different hUCMSCs treatment groups. Data are presented as mean ± SD. ^###^P < 0.001 vs control group; ^*^P < 0.05 vs model group.

In the dose-effect relationship experiments, the brain tissues of rats in each group were stained with TTC 28 days after hUCMSCs administration (**Figures 10A–E**). The low-dose treatment group exhibited a slight reduction in the cerebral infarction area; however, no statistically significant value was observed. Meanwhile, the medium-dose treatment group and the high-dose treatment group could significantly reduce the cerebral infarction area. The three treatment groups improvement rates of cerebral infarction area were 15.8%, 21.7%, and 22.9%, respectively (**Figure 10F**). These results show that the middle and high doses of hUCMSCs had a higher effect on reducing the cerebral infarction area in MCAO rats.

## 4 Discussion

IS is a high-incidence disease with a wide time windows, different clinical manifestations at different onset periods, and targeted treatment requirements through different methods (30). In the acute phase (within 2 weeks), IS is mainly manifested as oxidative stress and neurotoxicity caused by the lack of cellular oxygen, glucose, and adenosine triphosphate (ATP) at the ischemic site, which in turn leads to increased cell damage and apoptosis (31). The reperfusion of blood flow after ischemia causes damage to the blood-brain barrier and blood vessels due to the secondary beating of damaged tissues and loss of vascular structural integrity, which further leads to cerebral edema and infarction (32). Unfortunately, there is no current effective treatment for brain tissue repair and functional recovery in the sub-acute and sequela phases of IS due to the inherent irreversibility of nerve cells (21).

hUCMSCs have been shown to return to the damaged site for tissue repair, promotion of endogenous neural cell repair after cerebral ischemia, effective inhibition of apoptosis in the ischemic penumbra, promotion of angiogenesis after cerebral ischemia, and inhibition of excessive inflammation through immune regulation (11, 33). Numerous preclinical studies and clinical reports on the use of stem cells in the treatment of IS have confirmed the safety and effectiveness of stem cells (20, 22, 34). However, studies exploring the mechanism and time windows of administration are relatively few, with fewer preclinical studies in the sub-acute and sequela phases of IS. Treatment methods that use stem cells with more comprehensive functions offer several advantages for diseases with strict treatment windows during the acute phase and limited treatment options.

In this study, we explored the underlying mechanism of injecting hUCMSCs at the acute phase of the disease and the time windows, as well as the dose-effect relationship of the injeciton at the acute, sub-acute, and sequelae phases of the disease. Since the brain tissues of acute stroke patients or animal models are severely damaged and the internal environment is poor during IS, treatment options should be given at a relatively later time windows. The expression of chemokines, neurotrophic factors, and angiogenic factors was increased in MCAO rats after the treatment in the acute stage. In the pre-clinical experiment of the time windows and dose-effect relationship, the results showed that under the same administration frequency, the earlier use of relatively high doses of hUCMSCs significantly increased the neurological and motor function scores, as well as increased the improvement rate of cerebral infarction area.

IS will activate the peripheral immune system in a short time after the blood-brain barrier is damaged, and the inflammatory cells will be overactivated and secrete a large amount of inflammatory factors into the blood (35). Damage to the blood-brain barrier leads to an increase in its permeability, and a large number of neutrophils, T cells, and macrophages infiltrate into the central nervous system.In this experiment, treatment with hUCMSCs effectively inhibits the activation of the peripheral immune system and regulates immune function. However, there is no treatment difference in the brain tissue has been observed. Based on this result, we speculate that hUCMSCs may not be able to enter the brain tissue for direct contact to participate in the treatment (36). We also used human nuclear staining antibodies to detect the brain tissue of MCAO rats 3 days and 5 days after injection of hUCMSCs. Consistent with published studies (22, 37), we did not find UCMSCs at this time window. We also considered using intracerebral injections, but this method resulted in increased mortality in MCAO rats.

hUCMSCs, under the action of the SDF-α/CXCR4 axis, may migrate to the blood-brain barrier and secrete neurotrophic factors through paracrine to inhibit the apoptosis of nerve cells in the brain tissue and promote the brain structure and function recovery (38, 39). However, the relatively poor therapeutic effect of hUCMSCs at a later time windows may be attributed to the liquefaction of damaged tissues and the formation of cysts that are not readily cleared by the body during the subacute and sequela stages of IS. Moreover, the inflammatory stimulation and the interference of the glial scar during this period may also limit stem cell function. In the future, more precise treatment can be carried out through gene modification, minimally invasive targeted implantation, specific differentiation, and other stem cell technologies, which is bound to improve the clinical significance of this treatment option.

## Data availability statement

The original contributions presented in the study are included in the article/**Supplementary Material**. Further inquiries can be directed to the corresponding author.

## Ethics statement

The studies involving human participants were reviewed and approved by Dongguan Changan Hospital Ethics Committee (2019507150102035). The patients/participants provided their written informed consent to participate in this study. The animal study was reviewed and approved by Youji (Tianjin) Pharmaceutical Technology Co., Ltd. Laboratory Animal Ethics Committee (IACUC20220325-04).

## Author contributions

NQ, HWW, and HW designed, directed and supervised the entire study. CJ, QLv, and QLiu, performed most of the experiments; FX, ZW, and HPZ analyzed all results; DS and HYZ wrote the manuscript. All authors contributed to the article and approved the submitted version.
