# Supplementary material for: Pre-clinical efficacy evaluation of human umbilical cord mesenchymal stem cells for ischemic stroke
Source: Front Immunol. 2023 Jan 13;13:1095469. doi: 10.3389/fimmu.2022.1095469 (PMC9885855; doi:10.3389/fimmu.2022.1095469)
Supplement: Supplementary file 1 [file Table_1.doc]

Table S1 Scoring criteria for the degree of neurological impairment in MCAO rats

| Item Performance Score | Item Performance Score | Item Performance Score |
| --- | --- | --- |
| 1.Exercise test | 1）Lift the rat's tail |  |
|  | Bend the forelimbs | 1 |
|  | Hind limb bending | 1 |
|  | Raise head >10. in 30s (with vertical axis) | 1 |
|  | 2) Placing the rat on the floor for walking (normal = 0; highest value = 3) |  |
|  | 0. Walking normally | 0 |
|  | 1. unable to walk straight | 1 |
|  | 2. turn in a circle toward the hemiplegic side | 2 |
|  | 3. leaning towards the hemiplegic side | 3 |
| 2.Crossbar balance test | Able to balance and have a stable posture | 0 |
|  | Grasps the side of the bar | 1 |
|  | Can hold the bar, but one limb is detached from the bar | 2 |
|  | Can hold the crossbar, but two limbs are detached from the crossbar, or on the crossbar | 3 |
|  | Attempted to balance on the crossbar (>40s), but fell off | 4 |
|  | Attempts to balance on the crossbar (>20s) but falls off | 5 |
|  | Shedding, unable to balance, or not wanting to struggle hanging from the crossbar | 6 |
| 3.Reflection test | Auricular reflex (shaking head when touching the ear canal opening) | 1 |
|  | Corneal reflex (blinking when touching the cornea lightly with cotton) | 1 |
|  | Panic reflex (brief sound of tearing paper produces motor response) | 1 |
|  | Seizures, myoclonus, dystonia | 1 |
| Total |  | 16 |
